# Omental patch as prevention for bile leak in patients undergoing subtotal cholecystectomy: a propensity score analysis

**DOI:** 10.1007/s13304-025-02129-w

**Published:** 2025-02-11

**Authors:** Camilo Ramírez-Giraldo, Violeta Avendaño-Morales, Alejandro González-Muñoz, Isabella Van-Londoño, Juan Felipe Díaz-Castrillón, Andrés Isaza-Restrepo

**Affiliations:** 1 Hospital Universitario Mayor–Méderi, Calle 24 #29–45, Bogotá, Colombia; 2 Universidad del Rosario, Bogotá, Colombia

**Keywords:** Cholecystectomy, Subtotal cholecystectomy, Laparoscopic, Cholecystitis, Bile leak

## Abstract

Subtotal cholecystectomy is one of the most frequent bail-out procedures performed during difficult cholecystectomy. A common complication to this procedure is bile leak, and thus multiple strategies have been created to avoid its appearance. This study aims to evaluate the effectivity of using an omental patch as bile leak prevention in patients undergoing subtotal cholecystectomy. A retrospective cohort study including patients who underwent subtotal cholecystectomy between 2014 and 2022 was performed. 17 patients had an omental patch, while 378 did not; the latter were included to evaluate surgical outcomes with bile leak as a primary outcome using a propensity score matching analysis (PSM). Patients’ median age in both groups after PSM was 71.00 (IQR: 59.00–81.00) and 69.00 (IQR: 61.75–80.25) years, respectively. The dominant sex in both groups was male. In most cases surgical procedure indication was cholecystitis. Patients who had an omental patch did not present statistically significant differences for bile leak rates compared to patients who did not (29.4% versus 17.6%, *p* = 0.456, respectively). Similar results were observed when evaluating the need for postoperative ERCP for bile leak management (23.5 versus 5.9%, *p* = 0.078). A statistically significant higher proportion of major complications were observed in patients who had an omental patch (47.1% versus 19.1%, *p* = 0.038). Pedicled omental patch was not an effective measure for preventing bile leak, and it even presented a higher rate of complications. It is thus imperative to continue evaluating other strategies for the prevention of bile leak during subtotal cholecystectomy.

## Background

Laparoscopic cholecystectomy is the procedure of choice for cholelithiasis and its complications [1–3]. Nonetheless, this procedure may prove difficult in cases in which it is not possible to complete the critical view of safety [4, 5]. As such, bailout procedures such as subtotal cholecystectomy are done in its stead to avoid bile duct injury and other complications [6–8]. Although subtotal cholecystectomy is one of the most frequently performed bailout procedures it is not exempt from complications including bile leak, retained stones, and subhepatic or subphrenic collections, among others [9–11].

Of the most common complications after subtotal cholecystectomy is bile leak, which can reach rates of up to 26.3% [10]. Documented risk factors for bile leak after subtotal cholecystectomy include fenestrating or “open-tract” cholecystectomy, acute cholecystitis, and surgery performed over 10 days after the initial procedure indication [12, 13]. The presentation of bile leak increases the length of hospital stay, the need for postoperative endoscopic retrograde cholangiopancreatography (ERCP), and the appearance of intraabdominal collections and fluid and electrolyte imbalances [13–15]. This considered, some procedures have been proposed so as to lower bile leak rates such as omental patches, falciform ligament patches and other devices, although these have limited evidence [16–20].

Considering the above, this study aims to evaluate the effectivity of the omental patch for bile leak prevention in patients undergoing subtotal cholecystectomy.

## Patients and methods

### Study design

An observational retrospective cohort study was designed. 395 subtotal cholecystectomies performed in our institution between 2014 and 2022 were reviewed. All variables were collected in an anonymous database. This study was reviewed and approved by our institution’s ethics committee (number DVO005 2349-CV1737). The study was conducted under the principles of the Declaration of Helsinki [21]. We followed the STROBE guidelines to report this study [22].

### Patients

Patients with a preoperative diagnosis of gallbladder cancer, patients in whom cholecystectomy was associated with another surgical procedure (such as gastrectomy or pancreatoduodenectomy), patients without 30 days postoperative follow-up, and patients whose data registry did not include the predetermined variables of interest were excluded.

The indications for performing laparoscopic cholecystectomy were the following: all cases of benign biliary disease (biliary cholic, pancreatitis, choledocholithiasis, cholecystitis, or a combination of them) where at least one diagnostic image study evidenced biliary disease. In cases of cholecystitis, this was diagnosed, classified, and managed following what is established in the Tokyo Guidelines [23, 24]. Additionally, the American Guidelines protocol for risk of choledocholithiasis was followed: in low-risk cases, cholecystectomy was performed without the need for additional studies; in intermediate-risk cases, a magnetic resonance cholangiopancreatography was performed; and in high-risk cases, patients were taken to an ERCP previous to performing cholecystectomy [25]. Cases of pancreatitis were managed according to both international and our institution’s guidelines, while the timing of cholecystectomy was defined as that when pancreatitis was clinically resolved [26]. All patients were assigned a follow-up appointment during the first postoperative month during which their general constitution, their surgical wounds, and the surgical specimen’s histopathological study were reviewed.

### Outcomes and covariates

The primary outcome measure was the presence of bile leak. Secondary outcomes were the requirement for postoperative ERCP, the presence of major complications according to the Clavien–Dindo score, hospital stay length, need for reintervention, and 30-day mortality.

The following variables were analyzed: patients’ demographic characteristics, body mass index, ASA Physical Status Classification, previous diagnosis of diabetes mellitus, arterial hypertension, chronic obstructive pulmonary disease, chronic kidney disease, or cardiovascular disease, Charlson comorbidity index, preoperative blood work tests, surgical procedure indication, imaging findings on preoperative image studies, classification of the severity of cholecystitis according to Tokyo Guidelines (in cases of cholecystitis), the need for preoperative ERCP, type of admission, time interval between hospital admission and surgical procedure, type of subtotal cholecystectomy according to Purzner’s classification [27], and intraoperative findings according to Nassar’s modified score for difficult cholecystectomy [5].

### Surgical procedure

**Fig. 1:**
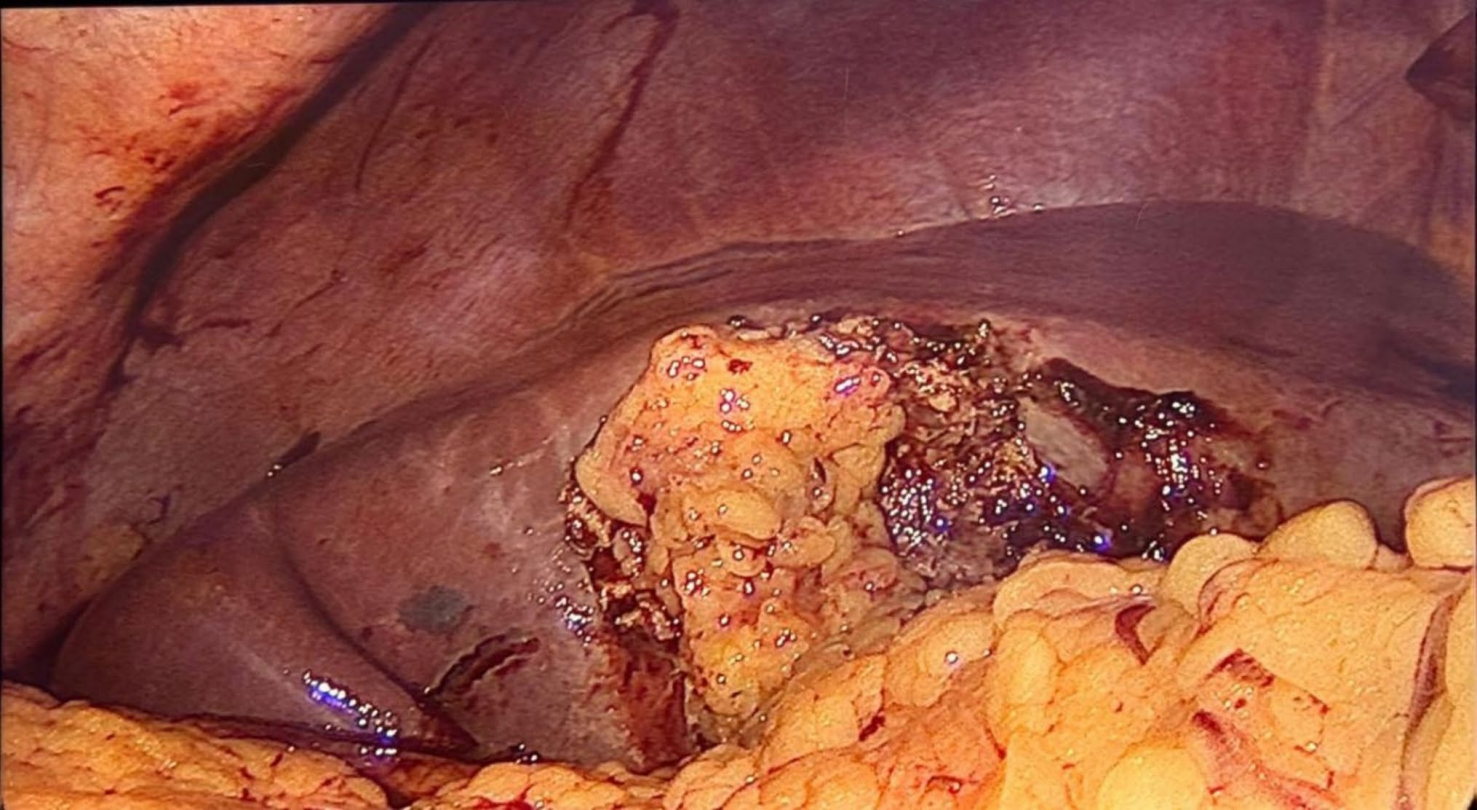
Pedicled omental patch in a subtotal cholecystectomy

Laparoscopic cholecystectomy was performed in the American position, using a four-port technique. Dissection of the hepatocystic triangle above the R4U safety line was performed. In cases where the critical view of safety could not be achieved and thus a safe cholecystectomy was not possible, the surgeon opted for a bailout procedure. When performing subtotal cholecystectomy as the bailout procedure, the attending surgeon tried to remove as much of the free side of the gallbladder wall as possible while staying above the safety line. The decision to remove the gallbladder wall in contact with the cystic plate was taken according to the attending surgeon’s own criterion. When it was not removed, the mucosa was treated with electrocautery. All gallstones were retrieved until the gallbladder’s infundibulum was reached, ensuring every stone had been removed. When the surgeon thought it was pertinent and if it was possible, the gallbladder stump was sutured. The omental segment where an omental patch was performed to avoid bile leak was always pedicled and not involved in the local inflammatory process. The decision to perform an omental patch was at the discretion of the surgeon performing the procedure. It was done by attaching the patch to the gallbladder walls using surgical sutures, accompanied by drain placement in the surgical site **(**Fig. 1**)**. When conversion to open cholecystectomy was performed, a midline or subcostal incision was done depending on the surgeon’s own preference while following the same steps as subtotal cholecystectomy using a laparoscopic approach [14, 15].

### Propensity score matching (PSM)

PSM analysis was performed to reduce selection bias and confounding factors in an observational study. Patients who underwent omental patch were matched with patients who did not undergo omental patch based on similar estimated propensity scores. Propensity scores were estimated using a logistic regression model that calculates the probability of an omental patch or non-assignment based on certain variables. Included variables were age, the Charlson comorbidity index, classification of severity of cholecystitis (a value of 0 indicates a diagnosis different from cholecystitis), previous ERCP, and type of subtotal cholecystectomy (reconstituting “closed-tract” or fenestrating “open-tract”). Propensity scores based on these variables made both groups’ characteristics comparable to equilibrate confounding factors between both groups and reduce selection bias. We excluded all participants who had missing data in one of the risk factors included in the PSM. We applied the nearest neighbor algorithm without replacement and set a caliper width at 0.2 of the logit of the standard deviation of the propensity score [28]. We identified 1:4 matching of exposed to unexposed participants as the optimal matching ratio based on the balance diagnosis after matching. Absolute standardized differences were used to assess the balance between groups after PSM [29].

Age as a variable was included in the PSM considering that there is a higher risk of complications and a higher rate of comorbidities as patients grow older. The Charlson comorbidity index was included for the same reasons [30]. The presence of cholecystitis and fenestrating or “open-tract” cholecystectomy has been documented as risk factors related to the appearance of bile leak [13]. On the other hand, a previous history of ERCP is related to less chances of presenting biliary fistula, probably due to a reduction in the pressure of Boyden’s sphincter complex [31].

A multivariable logistic regression model without matching was used as a sensitivity analysis, with bile leak as the outcome variable to check the robustness of our results after PSM. We adjusted for the same explanatory variables we included in the propensity score model.

### Statistical analyses

Baseline characteristics were reported by medians and continuous variables as IQRs. Frequencies and proportions were reported for dichotomous variables. A univariate analysis was performed with a *χ*^2^ value on categorical variables and with the Mann–Whitney test on continuous variables to compare the differences between variables according to whether omental patch was used or not, considering < 0.05 as a statistically significant p value. All analyses were carried out using R studio (version 2023.12.1 + 402).

## Results

**Fig. 2:**
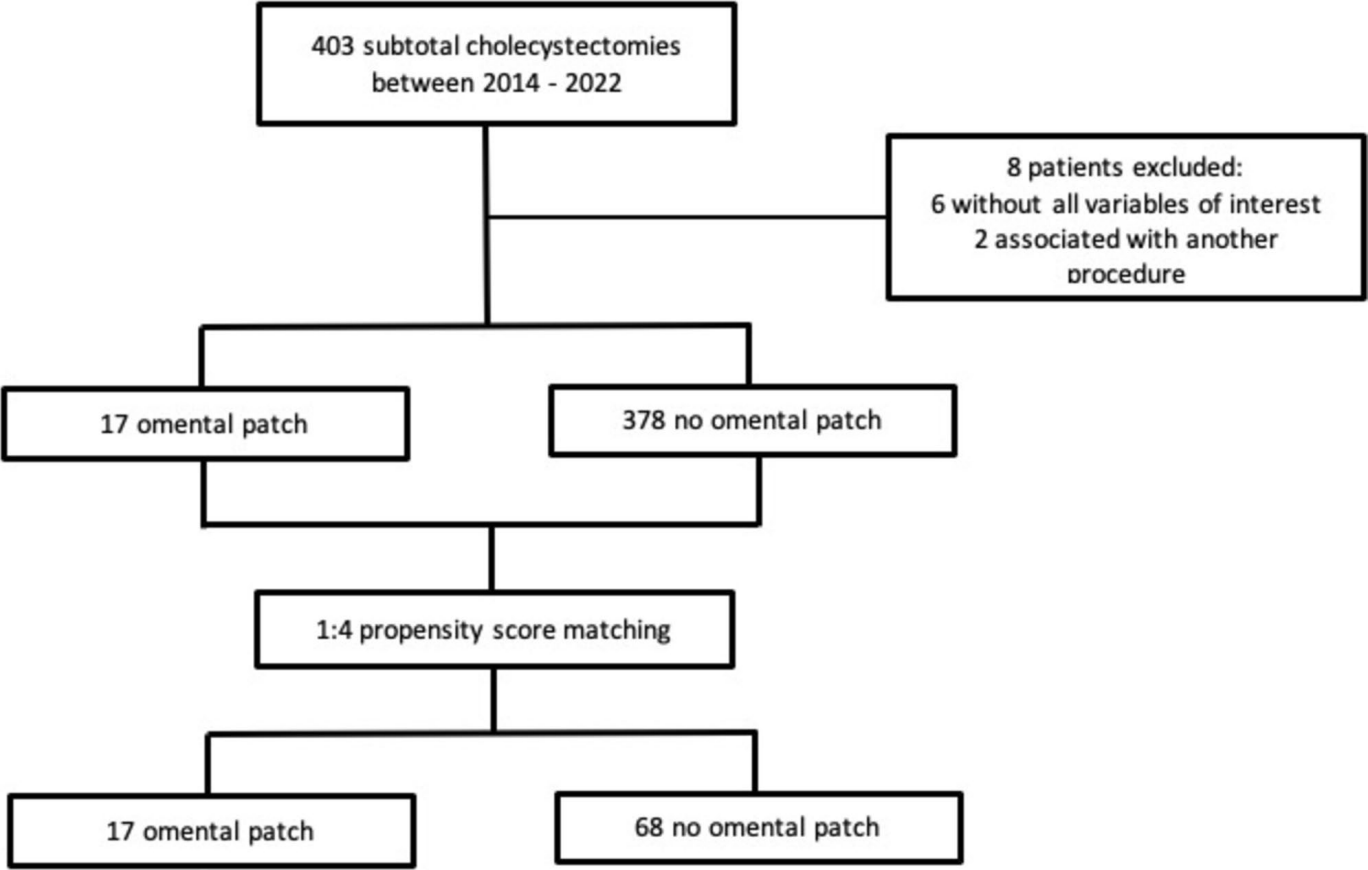
Study selection process flowchart

16,225 cholecystectomies were performed in our institution between 2014 and 2022. A total of 395 patients who underwent subtotal cholecystectomy were included in this study. 17 had an omental patch and 378 did not. The selection process can be seen in the following flowchart (Fig. 2).

The patients’ backgrounds before and after propensity score matching are shown in Table 1. After one-to-four matching, age, Charlson comorbidity index, classification of severity of cholecystitis, preoperative ERCP, and type of subtotal cholecystectomy were balanced. All absolute standardized differences were less than 0.1 after matching (Table 1**)**.

**Table 1:** Variables used for propensity score matching

Variables	Unmatched comparison			Matched comparison		
	Omental patch (*n* = 17)	No omental patch (*n* = 378)	Standardized difference	Omental patch (*n* = 17)	No omental patch (*n* = 68)	Standardized difference
Age (median)(IQR)(years)	71.00 (59.00–81.00)	68.00 (55.00–77.00)	0.5738	71.00 (59.00–81.00)	69.00 (61.75–80.25)	0.0243
Charlson comorbidity index (median)(IQR)(points)	3.00 [2.00, 5.00]	3.00 [1.00, 4.00]	0.2999	3.00 (2.00–5.00)	3.00 (2.00–4.00)	0.0316
Classification of severity of cholecystitis			0.1768			0.0738
0	4 (23.5)	90 (23.8)		4 (23.5)	14 (20.6)	
I	0 (0.0)	73 (19.3)		0 ( 0.0)	8 (11.8)	
II	11 (64.7)	162 (42.9)		11 (64.7)	29 (42.6)	
III	2 (11.8)	53 (14.0)		2 (11.8)	17 (25.0)	
Preoperative ERCP			0.1061			0.0000
No	14 (82.4)	296 (78.3)		14 (82.4)	56 (82.4)	
Yes	3 (17.6)	82 (21.7)		3 (17.6)	12 (17.6)	
Type of subtotal cholecystectomy			0.2054			0.0347
Fenestrating or “open-tract”	13 (76.5)	322 (85.2)		13 (76.5)	53 (77.9)	
Reconstituting or “closed-tract”	4 (23.5)	56 (14.8)		4 (23.5)	15 (22.1)	

The median age for patients in both groups after PSM was 71.00 (IQR: 59.00–81.00) and 69.00 (IQR: 61.75–80.25) years, respectively. The dominant sex in both groups was male. In most cases, cholecystitis was the main surgical procedure indication. A statistically significant difference (*p* = 0.030) could be seen in the ASA classification between groups. Other demographic, clinical, and surgical characteristics after performing PSM can be seen in Table 2.

**Table 2:** Demographic, clinical, and surgical characteristics according to whether omental patch was used or not after PSM

	*N* (%)	Omental patch (*n* = 17)	No omental patch (*n* = 68)	*p* value
Age (median)(IQR)(years)	69.00 (61.00–81.00)	71.00 (59.00–81.00)	69.00 (61.75–80.25)	0.969*
Sex				0.472
Female	34 (60.0)	5 (29.4)	29 (42.6)	
Male	21 (40.0)	12 (70.6)	39(57.4)	
Body mass index (median)(IQR)(kg/m^2^)	26.00 (24.00–29.00)	26.40 (23.55–30.60)	26.10 (23.65–29.15)	0.723*
ASA classification				**0.030**
1	6 (9.7)	1 (6.2)	5 (10.9)	
2	32 (51.6)	12 (75.0)	20 (43.5)	
3	23 (37.1)	2 (12.5)	21 (45.7)	
4–5	1 (1.6)	1 (6.2)	0 (0.00)	
Co-morbidity				
Diabetes mellitus	26 (30.6)	6 (35.3)	20 (29.4)	0.860
Arterial hypertension	51 (60.0)	11 (64.7)	40 (58.8)	0.868
Chronic obstructive pulmonary disease	8 (9.4)	2 (11.8)	6 (8.8)	1.000
Chronic kidney disease	3 (3.6)	0 (0.0)	3 (4.4)	0.883
Cardiovascular disease	21 (24.7)	4 (23.5)	17 (25.0)	1.000
Charlson comorbidity index (median)(IQR)(points)	3.00 (2.00–4.00)	3.00 (2.00–5.00)	3.00 (2.00–4.00)	0.854*
Preoperative laboratories (mean)(SD)				
Leukocytes (× 10^3^)	12.54 (8.90–15.49)	12.82 (10.44–14.58)	12.46 (8.47–15.49)	0.559*
Hemoglobin (mg/dL)	14.60 (13.20–16.00)	15.10 (13.15–16.35)	14.50 (13.20–15.85)	0.371*
Bilirubin (mg/dL)	0.90 (0.60–2.20)	1.01 (0.90–2.00)	0.91 (0.52–2.19)	0.429*
Imaging findings				
Bile duct diameter (mean)(SD)(mm)	5.00 (5.00–6.00)	5.00 (5.00–5.00)	5.00 (5.00–6.00)	0.739*
Gallbladder wall thickness (mean)(SD)(mm)	4.00 (2.00–4.00)	4.00 (2.00–4.00)	4.00 (2.00–4.00)	0.916*
Surgical procedure indication				
Biliary colic	11 (12.9)	2 (11.8)	9 (13.4)	1.000
Pancreatitis	5 (5.9)	2 (11.8)	3 (4.5)	0.575
Choledocholithiasis	11 (12.9)	0 (0.0)	11 (16.4)	0.165
Cholecystitis	67 (78.8)	13 (76.5)	54 (79.4)	1.000
Classification of severity of cholecystitis				0.210
I	8 (11.9)	0 (0.0)	8 (11.8)	
II	40 (59.7)	11 (64.7)	29 (42.6)	
III	19 (28.4)	2 (11.8)	17 (25.0)	
Preoperative ERCP				1.000
No	70 (82.4)	14 (82.4)	56 (82.4)	
Yes	15 (17.6)	3 (17.6)	12 (17.6)	
Type of admission				0.418
Elective	3 (3.5)	1 (5.9)	2 (2.9)	
Delayed	77 (90.6)	14 (82.4)	63 (92.6)	
Emergency	5 (5.9)	2 (11.8)	3 (4.4)	
Time from admission to surgical procedure (mean)(SD)(days)	5.00 (2.00–6.00)	5.00 (3.00–6.00)	4.50 (2.00–7.00)	0.860*
Type of subtotal cholecystectomy				1.000
Fenestrating or “open-tract”	66 (77.6)	13 (76.5)	53 (77.9)	
Reconstituting or “closed-tract”	19 (22.4)	4 (23.5)	15 (22.1)	
Nassar classification				0.307
I	0 (0.0)	0 (0.0)	0 (0.0)	
II	2 (2.4)	1 (5.9)	1 (1.5)	
III	7 (8.3)	0 (0.0)	7 (10.4)	
IV	22 (26.2)	6 (35.3)	16 (23.9)	
V	53 (63.1)	10 (58.8)	43 (64.2)	

*p* values were obtained using the Chi-squared test. Bold values indicate statistically significant *p* values (*p* < 0.05)

*p values were obtained using the two-tailed *t* test

Patients who had an omental patch did not present statistically significant difference in bile leak proportions when compared to patients who did not have an omental patch (29.4% versus 17.6%, *p* = 0.456). Similar results were observed when evaluating the postoperative need for ERCP as bile leak management (23.5 versus 5.9%, *p* = 0.078). However, it is important to highlight that these proportions were nonetheless higher in patients who had an omental patch. When evaluating major complications (defined as a Clavien–Dindo score > 3), a statistically significant higher rate of presentation was observed in patients who had an omental patch (47.1 versus 19.1, *p* = 0.038), as well as presenting longer hospital stays (12 days versus 9 days, *p* = 0.008). Moreover, both mortality and reintervention had higher rates in the group of patients who had an omental patch, although without statistically significant differences (Table 3).

**Table 3:** Surgical outcomes according to whether omental patch was used or not after PSM

	*N* (%)	Omental patch (*n* = 17)	No omental patch (*n* = 68)	*p* value
Hospital stay (median)(IQR)(days)	9.00 (1.00–13.00)	12.00 (9.00, 20.00)	9.00 (6.00, 12.00)	0.008*
ICU stay (median)(IQR)(days)	0.00 (0.00–1.10)	0.00 (0.00–2.00)	0.00 (0.00–0.00)	0.195*
Bile leak				0.456
No	68 (80.0)	12 (70.6)	56 (82.4)	
Yes	17 (20.0)	5 (29.4)	12 (17.6)	
Postoperative ERCP				
Choledocholithiasis	1 (1.2)	0 (0.0)	1 (1.5)	1.000
Bile leak	8 (9.4)	4 (23.5)	4 (5.9)	0.078
Complications				
Bile duct injury	2 (2.4)	0 (0.0)	2 (2.9)	1.000
Bilioperitoneum	1 (1.2)	1 (5.9)	0 (0.0)	0.451
Intestinal injury	1 (1.2)	0 (0.0)	1 (1.5)	1.000
Wound infection	3 (3.5)	1 (5.9)	2 (2.9)	1.000
Fluid collection	0.0 (0.000–0.00)	0 (0.0)	1 (1.5)	1.000
Perioperative acute myocardial infarction	1 (1.2)	0 (0.0)	0 (0.0)	
Perioperative venous thromboembolism	0 (0.0)	1 (5.9)	0 (0.0)	0.451
Health care-associated pneumonia	1 (1.2)	0 (0.0)	0 (0.0)	0.564
Health care-associated urinary tract infection	0 (0.0)	2 (11.8)	3 (4.4)	
Pleural effusion	5 (5.9)			
Reintervention				0.169
No	79 (92.9)	14 (82.4)	65 (95.6)	
Yes	6 (7.1)	3 (17.6)	3 (4.4)	
Major complication (Clavien–Dindo ≥ III)				**0.038**
No	64 (72.3)	9 (52.9)	55 (80.9)	
Yes	21 (24.7)	8 (47.1)	13 (19.1)	
30-day mortality				0.186
No	82 (96.5)	15 (88.2)	67 (98.5)	
Yes	3 (3.5)	2 (11.8)	1 (1.5)	

*p* values were obtained using the Chi-squared test. Bold values indicate statistically significant p values (*p* < 0.05)

*p values were obtained using the two-tailed *t* test

A similar trend was observed in the sensitivity analysis results for bile leak with omental patch placement, albeit with an elevated effect before the PSM (OR = 2.22, 95% CI 2.25–4.67, *p* =  < 0.001) compared with after the PSM (OR = 1.92, 95% CI 0.93–4.05, *p* = 0.081). In none of these cases was omental patch placement effective for preventing bile leak presentation.

## Discussion

In this retrospective cohort study, we evaluated the efficacy of omental patch as a preventive measure for bile leak in patients undergoing subtotal cholecystectomy. Our findings revealed no significant reduction in bile leak incidence when employing an omental patch compared to cases that did not. Interestingly, patients who received an omental patch had significantly longer hospital stays and a higher rate of major complications. There was no significant difference in the need for postoperative ERCP due to bile leak or 30-day mortality between both groups, although the overall rates were higher in the group that had an omental patch. These results may be influenced by selection bias, as the omental patch might have been used more frequently in patients with more severe intraoperative inflammation—an aspect that the Tokyo classification used in the PSM analysis does not fully account for. Consequently, while the omental patch does not appear to reduce bile leak rates, its use is associated with increased hospital stays and a higher incidence of major complications, potentially reflecting the complexity and severity of the cases in which it was employed.

Current procedures described in the literature for bile leak prevention during subtotal cholecystectomy are scarce; for example, performing a reconstituting or “closed-tract” type subtotal cholecystectomy is associated with a lower incidence of bile leak and other complications including wound infection, reintervention, need for ERCP, and subhepatic or phrenic collections, and as such it is the procedure of choice when its execution is feasible. Nonetheless, a recent study evidenced that subtotal fenestrating or “open-tract” laparoscopic cholecystectomy yields better surgical outcomes including lower bile duct injury rates along with shorter operative times and lower rates of bleeding, ICU admission, reoperation, and readmission, although with higher bile leak and ERCP rates. The authors of that study considered that the risk/benefit balance for fenestrating cholecystectomy deserves its placement as the bailout procedure of choice during subtotal cholecystectomy [32]. However, the study failed to evaluate complications following ERCP, which are critical data considering that it is an invasive procedure and that 43% of the subtotal laparoscopic fenestrating or “open-tract” cholecystectomies underwent this intervention [32, 33].

Some of the reasons for avoiding reconstituting or “closed-tract” subtotal laparoscopic cholecystectomy include longer operation time and its technical difficulty. With this in mind, a gallbladder closure system using LapraTy^®^ that was then covered with a pedicled omental segment was recently described, reporting six successful cases that did not present bile leak [19]. Additionally, some studies have also described successful cases where a pedicled omental patch is used to prevent bile leak [16]. It is also important to highlight that the pedicled omental patch can be employed in either reconstituting or “closed-tract” and fenestrating or “open-tract” type cholecystectomies, as both procedures present the risk of biliary fistula appearance.

In a study by Matsu et al., a non-pedicled omental patch was employed to avoid bile leak, reporting a lower rate of bile leak when compared to patients who did not have an omental patch (6% versus 44%). The reasoning behind using a non-pedicled versus a pedicled omental segment is the following: “First, dorsal vision of the stump is obstructed by the pedicle. Second, suturing cuts off the blood supply to the plugged omental tip. Third, there is little normal, soft omentum surrounding the gallbladder” [20]. Despite its limitations due to the small sample size, the fact that this technique has not been further evaluated stands out considering its initially promising results.

The use of a falciform ligament patch for bile leak prevention during fenestrating or “open-tract” cholecystectomy has also been described; however, there have not been any additional studies that evaluate its efficacy [17]. Other evaluated alternatives include occluding the cystic duct with cyanoacrylate glue to prevent bile leak during fenestrating cholecystectomy, but this procedure is not recommended because the glue can migrate to the bile duct and cause bile duct obstruction [15, 18].

The lack of effectivity for the pedicled omental patch in this study could be attributed to selection bias that may not have been properly controlled despite performing a PSM, or due to Matsui et al.’s arguments for employing a non-pedicled omental patch [20], or simply because the pedicled omental patch is not effective in preventing bile leak. Some of the limitations for this study include its retrospective nature and the limited sample size.

Considering the growing incidence of subtotal cholecystectomy as the preferred bailout procedure during difficult cholecystectomy [34, 35], it is imperative to perform higher quality studies to obtain better evidence on risk factors, prevention, and management of its complications as we become more frequently challenged by them.

## Conclusions

Pedicled omental patch was not an effective measure for bile leak prevention and was even associated with a higher rate of complications. It is necessary to continue evaluating other strategies for bile leak during subtotal cholecystectomy. Additionally, a high-quality multicenter prospective cohort study or clinical trial is required to clarify the indications for the omental patch.

## Data Availability

Data are available in https://doi.org/10.34848/LP51IE.
